# Topology Universality and Dissimilarity in a Class of Scale-Free Networks

**DOI:** 10.1371/journal.pone.0161653

**Published:** 2016-08-25

**Authors:** Lanhua Zhang, Juan Chen, Mei Wang, Yujuan Li, Shaowei Xue, Yiyuan Tang, Baoliang Sun

**Affiliations:** 1 College of Information and Engineering, Taishan Medical University, Taian 271016, China; 2 Center for Cognition and Brain Disorders, Department of Psychology, Hangzhou Normal University, Hangzhou 310036, China; 3 Department of Psychological Sciences, Texas Tech University, TX 79409, United States of America; 4 Key Lab of Cerebral Microcirculation in Universities of Shandong, Taishan Medical University, Taian 271016, China; 5 Department of Neurology, Affiliated Hospital of Taishan Medical University, Taian 271000, China; Lanzhou University of Technology, CHINA

## Abstract

We study the effect of subtle changes on the evolution in the scale-free (SF) networks. Three extended models are evolved based on competition and inner anti-preferential deletion in growth and preferential attachment processes. By nonlinear and dynamic controlling on randomness and determinacy, three models can self-organize into scale-free networks, and diverse scaling exponents appear. Moreover, the model with more determinacy has more stringent parameter control than randomness, especially in the edge deletion. Our results suggest that the nature of the topology universality and dissimilarity in SF networks may be the subtle changes of randomness and determinacy.

## Introduction

Newton and Einstein all said that the nature is simple. The emergence of complex networks make people pay more attention to the interaction of networks corresponding to the real world [1–5]. What are the universality and dissimilarity if we look on the real world as interaction network? How does the real world evolve from simple to complex, and what are the evolution processes? How does the real world change when it is subjected to external forces?

Is the evolution completely random or artificially controlling, or both coexist? To answer these questions, we probe into the evolutions of the complex network systems [2]. Meanwhile, what are their roles in the evolutions as the two important factors in complex networks of randomness and regularity [3]? How does it evolve with randomness and regularity for the real network of large scale? Is it strictly planning or completely random, or both coexist?

About the universality and dissimilarity of evolving networks [6], researchers have been searching the key factors for the questions so as to serve the real world quickly and easily. In order to explore the universality and dissimilarity in complex network evolutions, graph theory provides a unified tool to describe network structures [7], and nonlinear dynamics gradually develops to be one of the fundamental tools for the topology analysis of complex networks in order to find the interactions between the network behaviors [8].

To complex networks, at first in the 1960s, Erdös and Rényi put forward the random network model and set up the random graph theory [9]. Their analytical results are in line with the main characteristics of most real networks, so the random graph is the basic and direct selection for complex networks in the next 40 years. Since the small-world (SW) model [10] and SF model were demonstrated by Watts-Strogatz and Barabási-Albert (BA) [11] in 1998 and 1999 respectively, the characters of SW and SF to the real systems were revealed. The two big discoveries make people realize that large numbers of real networks are neither completely regular networks, nor entirely random networks.

It is well known that the clustering coefficient in regular network is higher and the average path length in random network is smaller that is in line with the characteristics of the actual systems by the analysis of regular networks and random networks [1, 9]. The results imply that the real systems are likely between regular networks and random networks, just like the chaos of the real world in the 1970s [12], which were the contradiction and unity relationship both the randomness and the certainty.

Since the real networks are the combination of random and regular characteristics, in fact, the evolution of SW model has indicated a transition from a fully regular network to a completely random network [10], whether do the results completely correspond to the real world evolving? However, Anishchenko [13] simulated an auto-associative memory network by ordered and random connectivity and found that the memory network tended to be mutually exclusive behaviors with random and regular, not simply related to the notion of SW. For the SF network model, Amaral regarded it as one of classes of SW networks [14], then whether are the randomness and the regularity coexisting in SF network, and how do they affect the topology of SF network?

In the real systems, random represents a common nature based on the results of random graph theory [9], however, all kinds of external control factors become more and more powerful with the development and expansion of the real systems so as to guarantee the healthy development. External control factors are expressed in many ways, such as determinacy [15], controllability [16] and stability [17]. Except that, researchers also put forward complexity [18, 19] and trade-off relationship [20] to study the topology characters for complex networks.

From the initial BA network [11] to the late evolution models, researchers have set up a large number of SF network models with a variety of evolution factors, such as random or deterministic factors combining with cost [14], aging [21], competition [22], power-hop and power-hop exponent [23] so as to look for the topology universality and dissimilarity.

In this paper, we still consider the factors of growth, preferential attachment, competition, aging, resource balance and control [11, 16, 17, 19–22] in order to evolve real world and find the key factors of the universality and dissimilarity for evolving networks. We evolve the complex network models by adjusting the deterministic or random factors and discuss the effect of determinacy and randomness on topology characters of SF networks based on our previous model [24].

## 1 The extended models

### 1.1 The model M

In our previous model [24], we optimized the SF model with competition and anti-preferential deletion so as to reflect the competition ability, aging degree, resource balance and control ability based on the mechanisms of growth and preferential attachment. For comparison, our optimized model is called model M where the evolution includes three steps, initialization, growth and optimization. Briefly as follows:

1. *Initialization*: Starting with *m*_0_ isolated nodes and then adding *m* new edges. Every edge is selected with random and preferential [11, 22, 24]. The preferential probability of the node *i* is
  $$\begin{array}{c} \prod \left(k_{i},\eta_{i}\right)=\frac{\eta_{i}k_{i}}{\sum_{j}\eta_{j}k_{j}} \end{array}$$ (1)
2. *Growth*: Adding a new node with *n* new edges. The node *i* wins the new edges by its probability ∏(*k*_*i*_, *η*_*i*_).
3. *Optimization*: Deleting *q* edges. The start and the end of the edges of the node *i* are deleted by its anti-preferential probability ∏*(*k*_*i*_, *η*_*i*_) [25],
  $$\begin{array}{c} \prod *\left(k_{i},\eta_{i}\right)=\frac{1}{N(t)-1}\left(1-\prod \left(k_{i},\eta_{i}\right)\right) \end{array}$$ (2)

### 1.2 The model R

Based on the model M, we evolve a new network model by increasing the randomness in order to explore the effect of the determinacy and the randomness on SF network in topology universality and dissimilarity, named model R for short. With the similar evolutionary processes, the model R begins with *m*_0_ isolated nodes, and at each time step comparing with the model M, (i) *Initialization* is the same but (ii) *Growth* is from competition to random, i.e., the node *i* wins the new edges randomly when a new node with *n* new edges is added. In (iii) *Optimization*, the deleting edges of the node *i* decrease the determinacy, that is, with random and anti-preferential probability ∏*(*k*_*i*_, *η*_*i*_).

### 1.3 The model D

Similarly, we evolve the model D by increasing the determinacy. The model D has the same processes of (ii) *Growth* and (iii) *Optimization* with the model M, but (i) *Initialization* improves the competition, i.e., a node *i* wins the start and the end points of a new edge all by its probability ∏(*k*_*i*_, *η*_*i*_) when adding *m* new edges to the model.

### 1.4 The model C

Similarly, we evolve the model C in order to improve the comparability. In the model C, (i) *Initialization* and (ii) *Growth* are the same as the model M. In (iii) *Optimization*, the deleting edges of the node *i* are selected by random and anti-preferential probability ∏*(*k*_*i*_, *η*_*i*_).

In all the models, *η*_*i*_ is the fitness [21] and *k*_*i*_ is the connectivity of the node *i*. *N*(*t*) is the size of the network and *m* ≥ 0, *m* ≤ *m*_0_, *n* > 0, *q* ≥ 0, *m* + *n* > *q*. Meanwhile, no nodes are reconnecting or self-join on the selection of the edge.

## 2 Degree distribution

By the discussion of the degree distribution *P*(*k*) in the model M [24], we got $P\left(k\right)=\frac{1}{A}\frac{{\left(n+\frac{B}{A}\right)}^{\frac{1}{A}}}{\left(k+\frac{B}{A}\right){}^{\frac{1}{A}+1}}$ and $1<\gamma =\frac{1}{A}+1\leq 3$.

### 2.1 Degree distribution of the model R

With the same processes by the continuum theory [11], the connectivity *k*_*i*_ is the sum of three parts, initialization, growth and optimization. The expressions are as follows:

$$\begin{array}{c} {\left(\frac{\partial k_{i}}{\partial t}\right)}_{\left(i\right)}=m\frac{1}{N}+m\left(1-\frac{1}{N}\right)\frac{\eta_{i}k_{i}}{\sum_{j}\eta_{j}k_{j}} \end{array}$$ (3)

$$\begin{array}{c} {\left(\frac{\partial k_{i}}{\partial t}\right)}_{\left(ii\right)}=n\frac{1}{N} \end{array}$$ (4)

$$\begin{array}{c} {\left(\frac{\partial k_{i}}{\partial t}\right)}_{\left(iii\right)}=-q\left[\frac{1}{N}+\left(1-\frac{1}{N}\right)\prod^{*}\left(k_{i}\right)\right] \end{array}$$ (5)

$$\begin{array}{c} \frac{\partial k_{i}}{\partial t}=\frac{m+n-3q}{N}+\left(m+\frac{q-m}{N}\right)\frac{\eta_{i}k_{i}}{\sum_{j}\eta_{j}k_{j}} \end{array}$$ (6)

The network size *N* is *N*(*t*) = *m*_0_ + *t* with time *t* goes on and *m*_0_ + *t* ≈ *t* for the large *t* [24], thus

$$\begin{array}{c} \frac{\partial k_{i}}{\partial t}\approx \frac{m+n-3q}{t}+\left(m+\frac{q-m}{t}\right)\frac{\eta_{i}k_{i}}{\sum_{j}\eta_{j}k_{j}} \end{array}$$ (7)

We know that ∑_*j*_ *η*_*j*_ *k*_*j*_ approximates to *C*(*m* + *n* − *q*)*t* for the large *t* from the references [22, 24], thus

$$\begin{array}{c} \frac{\partial k_{i}}{\partial t}\approx \frac{m+n-3q}{t}+\frac{m\eta_{i}}{C(m+n-q)t}k_{i}+\frac{\left(q-m\right)\eta_{i}}{C\left(m+n-q\right)t^{2}}k_{i} \end{array}$$ (8)

$\frac{1}{t^{2}}$ can be neglected for the large *t*, so

$$\begin{array}{c} \frac{\partial k_{i}}{\partial t}\approx \frac{m+n-3q}{t}+\frac{m\eta_{i}}{C(m+n-q)t}k_{i} \end{array}$$ (9)

The expression becomes a unified format by the same transformation and similar definition with $A=\frac{m}{C(m+n-q)}\eta_{i}$ and *B* = *m* + *n* − 3*q* from the model M [24],

$$\begin{array}{c} \frac{\partial k_{i}}{\partial t}-\frac{A}{t}k_{i}=\frac{B}{t} \end{array}$$ (10)

We get the same form of degree distribution by the same analysis and computations in the model M from (17) to (26) in the reference [24], that is,

$$\begin{array}{c} P\left(k\right)=\frac{\partial (k_{i}<k)}{\partial k}=\frac{t}{m_{0}+t}\frac{1}{A}\frac{{\left(n+\frac{B}{A}\right)}^{\frac{1}{A}}}{\left(k+\frac{B}{A}\right){}^{\frac{1}{A}+1}} \end{array}$$ (11)

We get

$$\begin{array}{c} P\left(k\right)\approx \frac{1}{A}\frac{{\left(n+\frac{B}{A}\right)}^{\frac{1}{A}}}{\left(k+\frac{B}{A}\right){}^{\frac{1}{A}+1}} \end{array}$$ (12)

### 2.2 Degree distribution of the model D

By the similar analysis of the model D with the model R, we get

$$\begin{array}{c} {\left(\frac{\partial k_{i}}{\partial t}\right)}_{\left(i\right)}=m\frac{\eta_{i}k_{i}}{\sum_{j}\eta_{j}k_{j}}+m\left(1-\frac{\eta_{i}k_{i}}{\sum_{j}\eta_{j}k_{j}}\right)\frac{\eta_{i}k_{i}}{\sum_{j}\eta_{j}k_{j}} \end{array}$$ (13)

$$\begin{array}{c} {\left(\frac{\partial k_{i}}{\partial t}\right)}_{\left(ii\right)}=n\frac{\eta_{i}k_{i}}{\sum_{j}\eta_{j}k_{j}} \end{array}$$ (14)

$$\begin{array}{c} {\left(\frac{\partial k_{i}}{\partial t}\right)}_{\left(iii\right)}=-q\left[\prod^{*}\left(k_{i}\right)\times 1+\sum_{j\neq i}\prod^{*}\left(k_{j}\right)\prod^{*}\left(k_{i}\right)\right] \end{array}$$ (15)

$$\begin{array}{c} \frac{\partial k_{i}}{\partial t}=-\frac{2q}{N-1}+\left(2m+n+\frac{2q}{N-1}\right)\frac{\eta_{i}k_{i}}{\sum_{j}\eta_{j}k_{j}}-m{\left(\frac{\eta_{i}k_{i}}{\sum_{j}\eta_{j}k_{j}}\right)}^{2}+q{\left(\frac{1-\frac{\eta_{i}k_{i}}{\sum_{j}\eta_{j}k_{j}}}{N-1}\right)}^{2} \end{array}$$ (16)

$$\begin{array}{c} \frac{\partial k_{i}}{\partial t}\approx -\frac{2q}{t}+\frac{\left(2m+n\right)\eta_{i}}{C(m+n-q)t}k_{i} \end{array}$$ (17)

The expressions become the unified form Eq (10) and degree distribution form Eq (12) given $A=\frac{2m+n}{C(m+n-q)}\eta_{i}$ and *B* = −2*q*.

### 2.3 Degree distribution of the model C

By the similar analysis processes, we obtain

$$\begin{array}{c} {\left(\frac{\partial k_{i}}{\partial t}\right)}_{\left(i\right)}=m\frac{1}{N}+m\left(1-\frac{1}{N}\right)\frac{\eta_{i}k_{i}}{\sum_{j}\eta_{j}k_{j}} \end{array}$$ (18)

$$\begin{array}{c} {\left(\frac{\partial k_{i}}{\partial t}\right)}_{\left(ii\right)}=n\frac{\eta_{i}k_{i}}{\sum_{j}\eta_{j}k_{j}} \end{array}$$ (19)

$$\begin{array}{c} {\left(\frac{\partial k_{i}}{\partial t}\right)}_{\left(iii\right)}=-q\left[\frac{1}{N}+\left(1-\frac{1}{N}\right)\prod^{*}\left(k_{i}\right)\right] \end{array}$$ (20)

$$\begin{array}{c} \frac{\partial k_{i}}{\partial t}=\frac{m-2q}{N}+\left(m+n+\frac{q-m}{N}\right)\frac{\eta_{i}k_{i}}{\sum_{j}\eta_{j}k_{j}} \end{array}$$ (21)

$$\begin{array}{c} \frac{\partial k_{i}}{\partial t}\approx \frac{m-2q}{t}+\frac{\left(m+n\right)\eta_{i}}{C(m+n-q)t}k_{i} \end{array}$$ (22)

We find that the model C is exactly the same with model M for the large time comparing the expression Eq (22).

## 3 Numerical simulation

We set different variable values to observe the changes of *P*(*k*) in different models in order to verify the correctness of theoretical prediction for degree distribution formula and demonstrate the degree distribution better. The setting basis of variable values refer to the constraint conditions.

From the processes of analysis in the models, we know that the actual and practical calculation formula expression is Eq (11), and the theoretical prediction of degree distribution formula is Eq (12).

**Fig 1:**
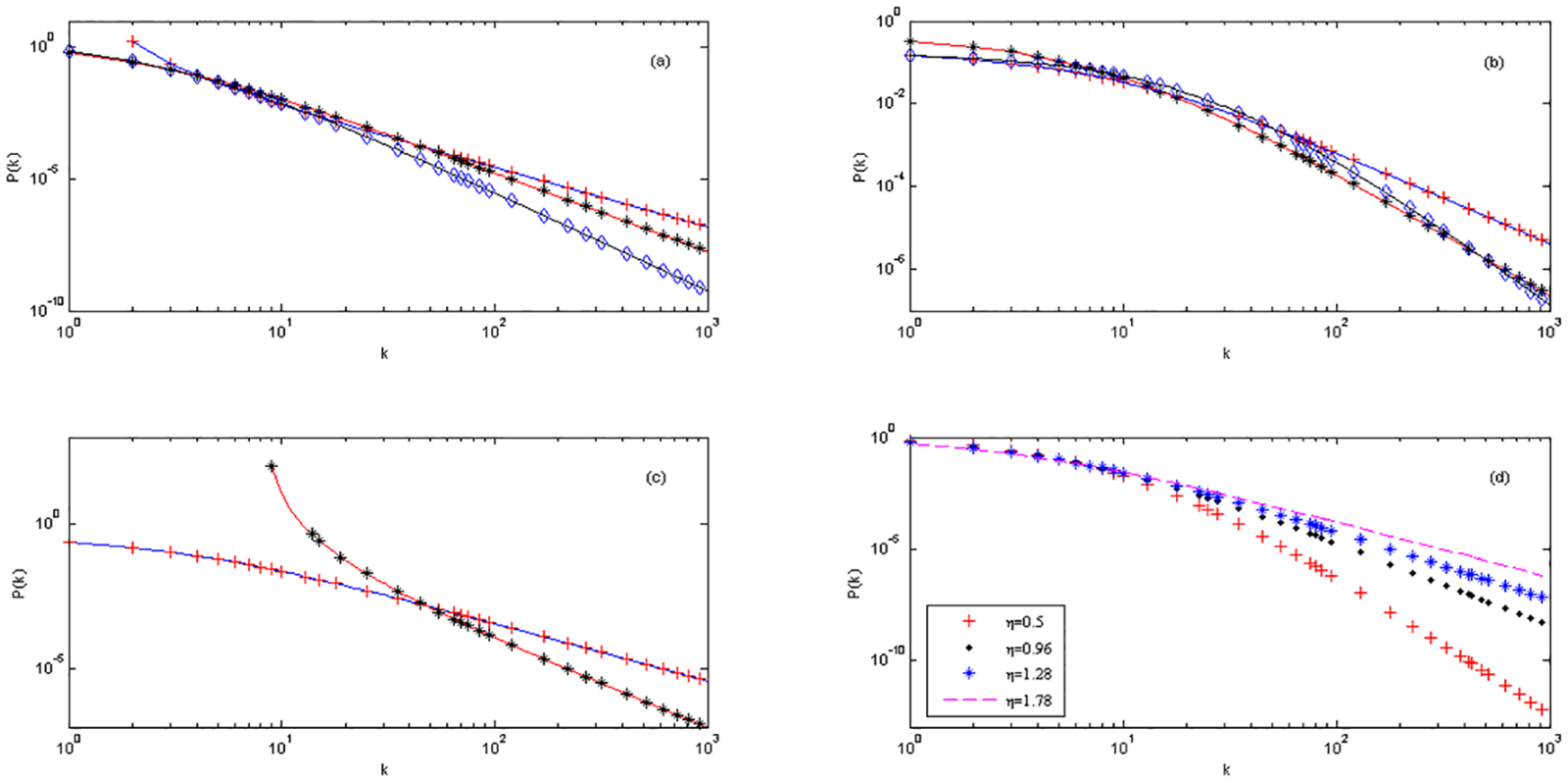
(a)—(c) With different conditions and parameters to compare the numerical simulation and the theoretical prediction of the degree distribution *P*(*k*) for the model R. (a) With the condition *q* = *n* and after time *t* = 1000, three groups of the parameters are $\frac{\eta }{C}=\frac{4}{5}$, *m*_0_ = *m* = 3, *q* = *n* = 2; $\frac{\eta }{C}=\frac{1}{2}$, *m*_0_ = *m* = 3, *q* = *n* = 1; $\frac{\eta }{C}=\frac{4}{11}$, *m*_0_ = *m* = 3, *q* = *n* = 2. Blue, red and black solid lines stand for the theoretical degree distribution *P*(*k*); red plus, black asterisk and blue diamond stand for the numerical simulation of degree distribution *P*(*k*) of three groups respectively. (b) With the condition *q* < *n* and after time *t* = 1000, three groups of the parameters are $\frac{\eta }{C}=\frac{7}{10}$, *m*_0_ = *m* = 8, *n* = 2, *q* = 1; $\frac{\eta }{C}=\frac{3}{4}$, *m*_0_ = *m* = 4, *n* = 3, *q* = 1; $\frac{\eta }{C}=\frac{1}{2}$, *m*_0_ = *m* = 8, *n* = 4, *q* = 1. Blue, red and black solid lines stand for the theoretical degree distribution *P*(*k*); red plus, black asterisk and blue diamond stand for the numerical simulation of degree distribution *P*(*k*) of three groups respectively. (c) With the condition *q* > *n* and after time *t* = 1000, two groups of the parameters are $\frac{\eta }{C}=\frac{1}{2}$, *m*_0_ = *m* = 4, *n* = 1, *q* = 3; $\frac{\eta }{C}=\frac{1}{4}$, *m*_0_ = *m* = 3, *n* = 1, *q* = 3. Blue and red solid lines stand for the theoretical degree distribution *P*(*k*); red plus and black asterisk stand for the numerical simulation of degree distribution *P*(*k*) of two groups respectively. (d) The numerical simulations of degree distribution *P*(*k*) with different competition abilities *η* and the static parameters *C* = 2, *m* = 3, *n* = 2, *q* = 1.

**Fig 2:**
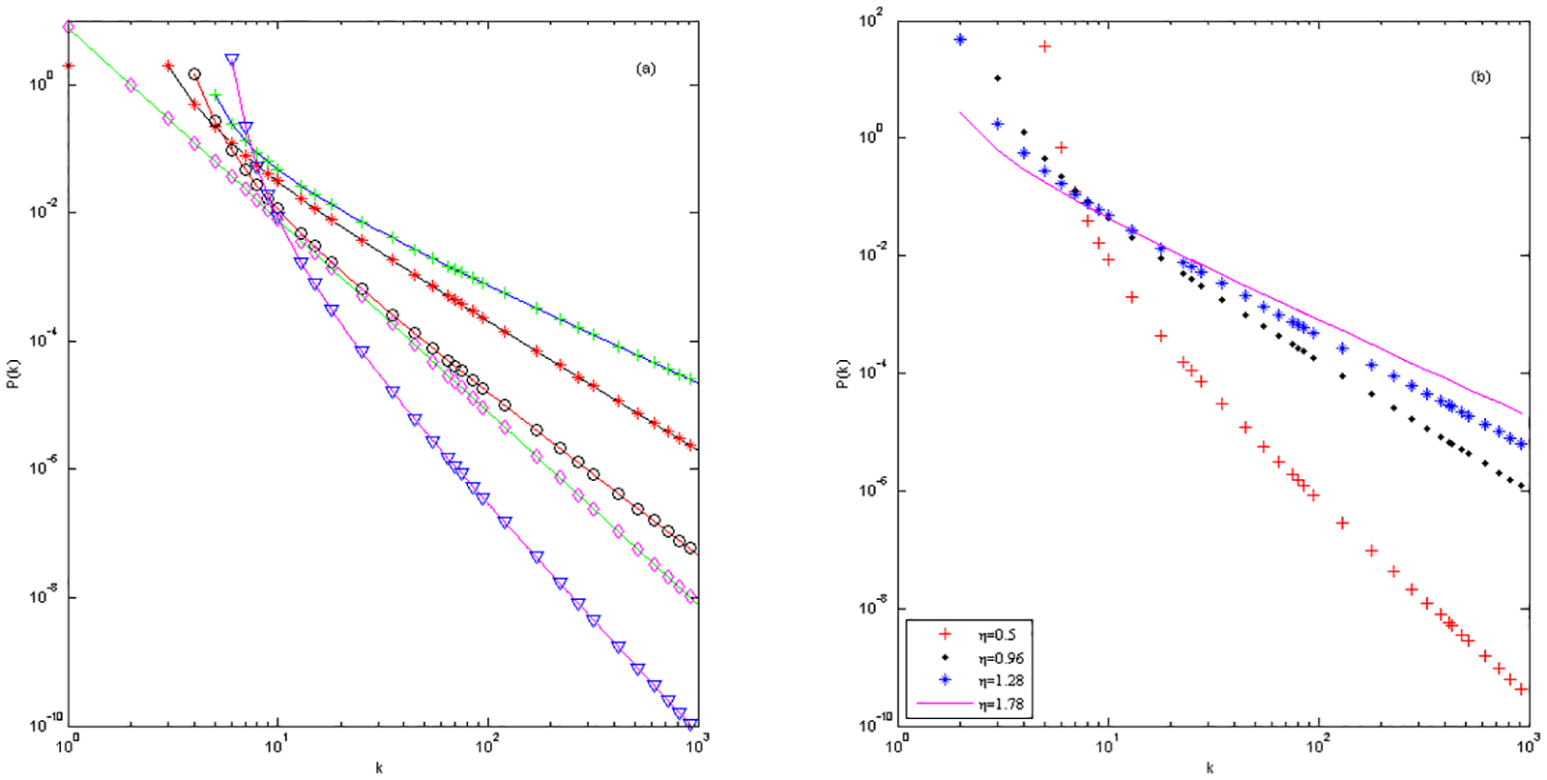
(a)—(b) With different parameters to compare the numerical simulations and the theoretical prediction of the degree distribution *P*(*k*) for the model D. (a) After time *t* = 1000, five groups of the parameters are $\frac{\eta }{C}=\frac{5}{6}$, *m*_0_ = *m* = 3, *n* = 6, *q* = 4; $\frac{\eta }{C}=\frac{4}{7}$, *m*_0_ = *m* = 5, *n* = 4, *q* = 1; $\frac{\eta }{C}=\frac{8}{21}$, *m*_0_ = *m* = 5, *n* = 4, *q* = 1; $\frac{\eta }{C}=\frac{1}{2}$, *m*_0_ = 3, *n* = 2, *m* = *q* = 0; $\frac{\eta }{C}=\frac{3}{13}$, *m*_0_ = *m* = 10, *n* = 6, *q* = 1;. Blue, black, red, green and fuchsine solid lines stand for the theoretical degree distribution *P*(*k*); green plus, red asterisk, black circle, fuchsine diamond and blue lower triangle stand for the numerical simulation of degree distribution *P*(*k*) of five groups respectively. (b) The numerical simulations of degree distribution *P*(*k*) with different competition abilities and the static parameters *C* = 2, *m* = 8, *n* = 5, *q* = 1.

Fig 1 shows the numerical simulation of the model R and Fig 2 shows the numerical simulation of the model D. The numerical simulation of the model C is just as the model M [24] because of the same expressions for the model C with the model M.

In Fig 1(a), three group situations are $A=\frac{4}{5}$, *B* = −1, $\gamma =\frac{9}{4}$; $A=\frac{1}{2}$, *B* = 1, *γ* = 3; $A=\frac{4}{11}$, *B* = 1, $\gamma =\frac{15}{4}$ respectively. In Fig 1(b), three group situations are $A=\frac{4}{5}$, *B* = 7, $\gamma =\frac{9}{4}$; $A=\frac{1}{2}$, *B* = 4, *γ* = 3; $A=\frac{4}{11}$, *B* = 9, $\gamma =\frac{15}{4}$ respectively. In Fig 1(c), two groups are *A* = 1, *B* = −4, *γ* = 2; $A=\frac{1}{2}$, *B* = −4, *γ* = 3 respectively. In Fig 1(d), the *B* = 2 and *A* and *γ* vary with *η*.

In Fig 2(a), five group situations are *A* = 2, *B* = −8, $\gamma =\frac{3}{2}$; *A* = 1, *B* = −2, *γ* = 2; $A=\frac{2}{3}$, *B* = −2, $\gamma =\frac{5}{2}$; $A=\frac{1}{2}$, *B* = 0, *γ* = 3; $A=\frac{2}{5}$, *B* = −2, $\gamma =\frac{7}{2}$ respectively. In Fig 2(b), the *B* = −2 and *A* and *γ* vary with *η*.

By the simulation results, we get that the degree distribution *P*(*k*) of numerical simulations are in good agreement with theoretical predictions for all the extended models.

## Discussion

From the expressions of all the extended models, the degree distribution formula Eq (12) has the same form of the model M [24], and is also similar to BA model, i.e., the extended models, the model R, the model D and the model C, all can self-organize into a kind of SF network.

Next we discuss the scaling exponent in all the extended models. We get the scaling exponent $\gamma =\frac{1}{A}+1$ from the degree distribution formula Eq (12) and *η*_*max*_ < *C* ≤ 2*η*_*max*_ by the results from the references [22, 24].

### 3.1 The model R

For every *i*, $\frac{1}{A}=\frac{m+n-q}{m}\frac{C}{\eta }$.

(1) If *η* = *η*_*max*_, $1<\frac{C}{\eta }\leq 2$. As *m* ≥ 0, *n* > 0, *q* ≥ 0, *m* + *n* > *q*, we have to discuss the relation between *n* and *q*.

(i) If *n* = *q*, $\frac{1}{A}=\frac{m+n-q}{m}\frac{C}{\eta }=\frac{C}{\eta }\in \left(1,2\right]$ and $2<\gamma =\frac{1}{A}+1\leq 3$. The result is consistent with other SF network models.

(ii) If *n* < *q*, as *m* + *n* > *q*, $\frac{m+n-q}{m}\in \left(0,1\right)$ and $\frac{1}{A}=\frac{m+n-q}{m}\frac{C}{\eta }\in \left(0,2\right)$, so $1<\gamma =\frac{1}{A}+1<3$. Given *γ* = 2:

If *C* = 2*η*_*max*_, $\frac{1}{A}=\frac{m+n-q}{m}\frac{C}{\eta }=1$, $\frac{m+n-q}{m}=\frac{1}{2}$. We get $q=n+\frac{m}{2}$. *γ* ∈ (1, 2) if $q>n+\frac{m}{2}$ and *γ* ∈ (2, 3) if $q<n+\frac{m}{2}$.

If $C=\frac{3}{2}\eta_{max}$, $\frac{m+n-q}{m}=\frac{2}{3}$. We get $q=n+\frac{m}{3}$. *γ* ∈ (1, 2) if $q>n+\frac{m}{3}$ and *γ* ∈ (2, 3) if $q<n+\frac{m}{3}$.

In these conditions, we infer that the bigger the *q* is, the more the scaling exponents fall into this range *γ* ∈ (1, 2), and the smaller the *q* is, the more the scaling exponents fall into this range *γ* ∈ (2, 3).

(iii) If *n* > *q*, $\frac{1}{A}=\frac{m+n-q}{m}\frac{C}{\eta }>1$ and $\gamma =\frac{1}{A}+1>2$.

If $C=\frac{7}{4}\eta_{max}$, we get *γ* = 3 when $q=n-\frac{5}{7}m$. *γ* ∈ (2, 3) if $n-\frac{5}{7}m<q<n$ and *γ* > 3 if $q<n-\frac{5}{7}m$.

If $C=\frac{5}{4}\eta_{max}$, we get *γ* = 3 when $q=n-\frac{7}{5}m$. *γ* ∈ (2, 3) if $n-\frac{7}{5}m<q<n$ and *γ* > 3 if $q<n-\frac{7}{5}m$.

In these conditions and given that the *q* is greater than 0, we infer that the bigger the *q* is, i.e., more close to *n*, the more the scaling exponents fall into this range *γ* ∈ (2, 3), and the smaller the *q* is, the more the scaling exponents will be away from 3, i.e., *γ* > 3.

(2) IF *η* < *η*_*max*_, as *η*_*max*_ < *C* ≤ 2*η*_*max*_, so $\frac{C}{\eta }>1$. Similarly we discuss the *γ* by the relation between *n* and *q*.

(i) If *n* = *q*, $\frac{1}{A}=\frac{C}{\eta }$, so $\frac{1}{A}>1$ and *γ* > 2. We get $\gamma =\frac{C}{\eta }+1$ and the *γ* depends on the $\frac{C}{\eta }$.

Given *γ* = 3, $\frac{C}{\eta }=2$ and *C* = 2*η*.

If $\eta =\frac{1}{2}\eta_{max}$, as *η*_*max*_ < *C* ≤ 2*η*_*max*_, $2<\frac{C}{\eta }\leq 4$ and 3 < *γ* ≤ 5. That is, *γ* > 3 if $\eta =\frac{1}{2}\eta_{max}$. If $\eta <\frac{1}{2}\eta_{max}$, $\frac{C}{\eta }$ and *γ* will be bigger, and the scaling exponents will be away from 3 with *η* being smaller and smaller. In this condition only $\eta >\frac{1}{2}\eta_{max}$, *γ* is likely *γ* = 3 or *γ* more falls into the range *γ* ∈ (2, 3).

(ii) If *n* < *q*, as *m* + *n* > *q*, $\frac{m+n-q}{m}>0$, so $\frac{1}{A}=\frac{m+n-q}{m}\frac{C}{\eta }>0$ and $\gamma =\frac{1}{A}+1>1$.

(a) Given *γ* = 3, $\frac{1}{A}=2$.

Given $\eta =\frac{1}{2}\eta_{max}$, $2<\frac{C}{\eta }\leq 4$.

If $\frac{C}{\eta }=4$, $q=n+\frac{m}{2}$. *γ* will be smaller if $q>n+\frac{m}{2}$, i.e., *γ* < 3, conversely *γ* will become bigger if $q<n+\frac{m}{2}$, i.e., *γ* > 3.

If $\frac{C}{\eta }=\frac{5}{2}$, $q=n+\frac{m}{5}$. *γ* will be smaller if $q>n+\frac{m}{5}$, i.e., *γ* < 3, conversely *γ* will become bigger if $q<n+\frac{m}{5}$, i.e., *γ* > 3.

(b) Given *γ* = 2, $\frac{1}{A}=1$.

Given $\eta =\frac{1}{2}\eta_{max}$, $2<\frac{C}{\eta }\leq 4$.

If $\frac{C}{\eta }=4$, $q=n+\frac{3m}{4}$. *γ* will be smaller if $q>n+\frac{3m}{4}$, i.e., *γ* ∈ (1, 2), conversely *γ* will become bigger if $q<n+\frac{3m}{4}$, i.e., *γ* > 2.

If $\frac{C}{\eta }=\frac{5}{2}$, $q=n+\frac{3m}{5}$. *γ* will be smaller if $q>n+\frac{3m}{5}$, i.e., *γ* ∈ (1, 2), conversely *γ* will become bigger if $q<n+\frac{3m}{5}$, i.e., *γ* ∈ (2, 3).

From (a) and (b), under the condition of $\frac{C}{\eta }=4$, *γ* ∈ (1, 2) if $n+\frac{3m}{4}<q<m+n$; *γ* = 2 if $q=n+\frac{3m}{4}$; *γ* ∈ (2, 3) if $n+\frac{m}{2}<q<n+\frac{3m}{4}$; *γ* = 3 if $q=n+\frac{m}{2}$; *γ* > 3 if $n<q<n+\frac{m}{2}$. Under the condition of $\frac{C}{\eta }=\frac{5}{2}$, *γ* ∈ (1, 2) if $n+\frac{3m}{5}<q<m+n$; *γ* = 2 if $q=n+\frac{3m}{5}$; *γ* ∈ (2, 3) if $n+\frac{m}{5}<q<n+\frac{3m}{5}$; *γ* = 3 if $q=n+\frac{m}{5}$; *γ* > 3 if $n<q<n+\frac{m}{5}$. In these conditions, the scaling exponents will continue to change in the three intervals as long as *q* > *n*.

Meanwhile, $\frac{C}{\eta }$ will be bigger if $\eta <\frac{1}{2}\eta_{max}$. We get that the interval *γ* ∈ (2, 3) will be smaller, and the intervals *γ* ∈ (1, 2) and *γ* > 3 will be bigger by the same discussion similar to (a) and (b). Conversely, $\frac{C}{\eta }$ will be smaller if $\eta >\frac{1}{2}\eta_{max}$. We get that the interval *γ* ∈ (2, 3) will be bigger, and the intervals *γ* ∈ (1, 2) and *γ* > 3 will be smaller by the same discussion similar to (a) and (b).

(iii) If *n* > *q*, as $\frac{C}{\eta }>1$, $\frac{1}{A}=\frac{m+n-q}{m}\frac{C}{\eta }>1$ and $\gamma =\frac{1}{A}+1>2$.

Given *γ* = 3, $\frac{1}{A}=\frac{m+n-q}{m}\frac{C}{\eta }=2$.

Given $\eta =\frac{1}{2}\eta_{max}$, $2<\frac{C}{\eta }\leq 4$. The result of the expression $\frac{1}{A}=2$ is no solution because of $\frac{m+n-q}{m}>1$.

Given $\eta =\frac{3}{4}\eta_{max}$, $\frac{C}{\eta }\in \left(\frac{4}{3},\frac{8}{3}\right]$, but only the $\frac{C}{\eta }\in \left(\frac{4}{3},2\right]$ is possible because of $\frac{m+n-q}{m}>1$. That is, it is possible for $\frac{1}{A}=2$ only the $\frac{C}{\eta }<2$.

Given $\frac{C}{\eta }=\frac{5}{3}$, $q=n-\frac{m}{5}$ and *γ* = 3. *γ* will become smaller and more likely falls into the range *γ* ∈ (2, 3) when $q<n-\frac{m}{5}$, of course it will become bigger and more likely bigger than 3 when $q>n-\frac{m}{5}$, *γ*, i.e., *γ* > 3.

Given $\frac{C}{\eta }=\frac{3}{2}$, $q=n-\frac{m}{3}$ and *γ* = 3. *γ* will become smaller and more likely falls into the range *γ* ∈ (2, 3) when $q<n-\frac{m}{3}$, of course it will become bigger and more likely bigger than 3 when $q>n-\frac{m}{3}$, *γ*, i.e., *γ* > 3.

By the different values of $\frac{C}{\eta }$, we infer that the bigger the *η* is, the smaller $\frac{C}{\eta }$ is, and there will be a small range of *q* values that the scaling exponents fall into the range *γ* ∈ (2, 3), that is more stringent requirements for *q*. When the value of *q* is a little larger, the scaling exponents will be easy bigger than 3, i.e., *γ* > 3. Conversely, the smaller *η* is, the bigger $\frac{C}{\eta }$ is, and there will be a big range of *q* values that the scaling exponents fall into the range *γ* ∈ (2, 3), i.e., the value of *q* is relatively loose.

In these conditions and given that the *q* is greater than 0, we infer that the smaller the *q* is, the more the scaling exponents fall into the range *γ* ∈ (2, 3), and the bigger the *q* is, the more the scaling exponents will be away from 3, i.e., *γ* > 3.

If $\eta <\frac{1}{2}\eta_{max}$, $\frac{C}{\eta }$ will be easy bigger than 2 and $\frac{1}{A}=\frac{m+n-q}{m}\frac{C}{\eta }>2$, that is, *γ* > 3. Moreover, as *η* becomes smaller, the *γ* becomes larger.

### 3.2 The model D

For every *i*, $\frac{1}{A}=\frac{m+n-q}{2m+n}\frac{C}{\eta }$.

(1) If *η* = *η*_*max*_, $1<\frac{C}{\eta }\leq 2$. As *m* ≥ 0, *n* > 0, *q* ≥ 0, *m* + *n* > *q* and $\frac{m+n-q}{2m+n}=\frac{m+n-q}{m+n+m}\in \left(0,1\right]$, $\frac{1}{A}=\frac{m+n-q}{2m+n}\frac{C}{\eta }\in \left(0,2\right]$ and $1<\gamma =\frac{1}{A}+1\leq 3$.

Given *γ* = 3, *q* = *m* = 0.

Given *γ* = 2, $\frac{m+n-q}{2m+n}\frac{C}{\eta }=1$.

If $\frac{C}{\eta }=2$, $q=\frac{n}{2}$. *γ* ∈ (2, 3) if $q<\frac{n}{2}$ and *γ* ∈ (1, 2) if $q>\frac{n}{2}$.

If $\frac{C}{\eta }=\frac{6}{5}$, $q=\frac{4m-n}{6}$. *γ* ∈ (2, 3) if $q<\frac{4m-n}{6}$ and *γ* ∈ (1, 2) if $q>\frac{4m-n}{6}$.

In these conditions and given that the *q* is greater than 0, we infer that no matter how the value of $\frac{C}{\eta }$ changes, the bigger the *q* is, the more the scaling exponents fall into the range *γ* ∈ (1, 2), and the smaller the *q* is, the more the scaling exponents fall into the range *γ* ∈ (2, 3).

(2) If *η* < *η*_*max*_, $\frac{C}{\eta }>1$. As $\frac{m+n-q}{2m+n}\in \left(0,1\right]$, $\frac{1}{A}=\frac{m+n-q}{2m+n}\frac{C}{\eta }>0$ and $\gamma =\frac{1}{A}+1>1$.

Given *γ* = 2, $\frac{m+n-q}{2m+n}\frac{C}{\eta }=1$.

Given $\eta =\frac{1}{2}\eta_{max}$, $2<\frac{C}{\eta }\leq 4$.

If $\frac{C}{\eta }=4$, $q=\frac{3n}{4}+\frac{m}{2}$. The scaling exponents will be farther from 2 within the range of less than 2 if $q>\frac{3n}{4}+\frac{m}{2}$, i.e., *γ* ∈ (1, 2), conversely they will be more close to 2 within the range of less than 2 if $q<\frac{3n}{4}+\frac{m}{2}$, i.e., *γ* ∈ (1, 2).

If $\frac{C}{\eta }=\frac{5}{2}$, $q=\frac{3n+m}{5}$. The scaling exponents will be farther from 2 within the range of less than 2 if $q>\frac{3n+m}{5}$, i.e., *γ* ∈ (1, 2), conversely they will be more close to 2 within the range of less than 2 if $q<\frac{3n+m}{5}$, i.e., *γ* ∈ (1, 2).

Given *γ* = 3, $\frac{m+n-q}{2m+n}\frac{C}{\eta }=2$.

Given $\eta =\frac{1}{2}\eta_{max}$, $2<\frac{C}{\eta }\leq 4$.

If $\frac{C}{\eta }=4$, $q=\frac{n}{2}$. The scaling exponents will be farther from 3 within the range of less than 3 if $q>\frac{n}{2}$, i.e., *γ* ∈ (2, 3), conversely they will be more close to 3 within the range of less than 3 if $q<\frac{n}{2}$, i.e., *γ* ∈ (2, 3).

If $\frac{C}{\eta }=\frac{5}{2}$, $q=\frac{n-3m}{5}$. The scaling exponents will be farther from 3 within the range of less than 3 if $q>\frac{n-3m}{5}$, i.e., *γ* ∈ (2, 3), conversely they will be more close to 3 within the range of less than 3 if $q<\frac{n-3m}{5}$, i.e., *γ* ∈ (2, 3).

In these conditions by the different values of $\frac{C}{\eta }$, we infer that the bigger the *η* is, the smaller $\frac{C}{\eta }$ is, and there will be a small range of *q* values that the scaling exponents are close to the critical value 2 or 3, that is more stringent requirements for *q*. Conversely, the smaller the *η* is, the bigger $\frac{C}{\eta }$ is, and there will be a big range of *q* values that the scaling exponents are close to the critical value 2 or 3, i.e., the value of *q* is relatively loose.

Given *γ* > 3, $\frac{m+n-q}{2m+n}\frac{C}{\eta }>2$. The bigger the *q* is, the smaller $\frac{m+n-q}{2m+n}$ is because of $\frac{m+n-q}{2m+n}\in \left(0,1\right]$. Only the smaller the *q* is, the more likely the scaling exponents are to fall into the range from 2 to 3. Conversely, the bigger the *q* is, the more impossible the scaling exponents are to fall into the range from 2 to 3.

### 3.3 The model C

Comparing with the model M, there are subtle differences in their evolution although the expression $\frac{\partial k_{i}}{\partial t}$ and *P*(*k*) are the same for the large *t*. The difference is reflected in the deletion of the edge, that is *q* in ${\left(\frac{\partial k_{i}}{\partial t}\right)}_{\left(iii\right)}$. For the model M,

$$\begin{array}{c} {\left(\frac{\partial k_{i}}{\partial t}\right)}_{\left(iii\right)}=-q\left[\frac{2}{t-1}\left(1-\frac{\eta_{i}k_{i}}{\sum_{j}\eta_{j}k_{j}}\right)-{\left(\frac{1}{t-1}\left(1-\frac{\eta_{i}k_{i}}{\sum_{j}\eta_{j}k_{j}}\right)\right)}^{2}\right] \end{array}$$ (23)

For the model C, the randomness replaces part of the competition,

$$\begin{array}{c} {\left(\frac{\partial k_{i}}{\partial t}\right)}_{\left(iii\right)}=-q\left[\frac{1}{t}\left(2-\frac{\eta_{i}k_{i}}{\sum_{j}\eta_{j}k_{j}}\right)\right] \end{array}$$ (24)

Comparing the parameters of *q* in the two models, we infer that the ideal network state can quickly reach by the removal of a smaller edge in a competitive model, but it will come slower and need remove more edges in a randomness model.

For the model R and the model D, comparing the same parameters in the range *γ* ∈ (2, 3), we find that the value space of *q* is small in the model D, that is more strict for *q*, and in the model R, *q* is easier to come to *γ* ∈ (2, 3), that is looser for *q*. Based on the discussion, we infer that the network falls into the ideal range more quickly with the increase in the control of determinacy, and the value of *q* is needed smaller and smaller. Comparing to the real world of the network, it is more in line with the needs of rational use for resources in the actual system. Meanwhile, by the discussion of *η*, the smaller *η* is, the more unreasonable in accordance with the reality whether the model R or the model D.

Comparing to our four models from the randomness and determinacy, the strongest randomness is the model R where has the most extensive and average scale of exponents and is looser in the parameters. The strongest determinacy is the model D where has also the extensive scale of exponents, but the scaling exponents are easier to converge to *γ* ∈ (2, 3), meanwhile the model D is more strict in the parameters. The model C and the model M are in the middle of them, and the scale exponents are similar and have the average control degree in the parameters comparing with the mode R and the model D.

Bianconi [22] ever put forward that some restrictions affected the SF network topologies, and adjusted the power-law distribution and the scaling exponents by deterministic control with competition, which is an example leading to multiscaling in SF network of full competition. Albert [6] pointed that the universality of SF network, if exist, depended on the subtle changes of the network’s parameters, which is a capacity model of SF network with full random in processes. Except that, Chen [25] demonstrated two capacity and SF network models with the similar processes of the model R and the model D, but the results had a better scale of exponent which was full SF state except a small scale in the parameters. Overall, our models are in line with the results of SF networks researches.

For special cases, it is actually existing for *γ* ∈ (1, 2) or *γ* > 3. For example, the airplane network still needs to keep an site if the airplane site is in the military stronghold, even though it is not competitive, that is *η* ≪ *η*_*max*_ and *γ* > 3. Meanwhile, the state *n* ≤ *q* also can appear if the sum of resources is fixed.

Based on the discussion above, let’s focus on the topology universality and dissimilarity of SF evolving networks. From our model M [24], we know that the model M has a comprehensive and universal scale for the real world evolving. Then we get three different models by modifying the subtle changes in network evolution on three steps, and all the models can self-organize into SF state and have the same scale ranges with the model M, i.e., they are all more comprehensive and universal scale for the real world evolving. However, they present different parameter control abilities for the comprehensive and universal scales, despite the topology universality is similar. In particular, the effects on SF network topologies are different when we modify the determinacy and randomness in evolving, i.e., topology dissimilarity is affected by some subtle changes, meanwhile, the degrees of dissimilarity are not the same.

By the topology universality and dissimilarity in SF network models, we can conclude that the subtle changes on randomness and determinacy affect the nature of the topology universality and dissimilarity, and the change is nonlinear and dynamic.

## Conclusion

Summary of our models and other SF models, many networks in real world can eventually evolve into SF state as long as following the growth and preferential attachment, regardless of the middle process of random or deterministic, including a variety of competition, aging, optimization, cost, capacity, controllability, stability, trade-off relationship and local events.

From the the complex network developments we know that the control of determinacy is becoming more and more important in the evolutions although the randomness is in line with the characteristics of most of the real network evolutions, especially in SW networks and SF networks. From our models, we know that the ideal state comes more quickly and less cost by deterministic control although the model R can also eventually evolve to an ideal state and will be more cost in *n* ≤ *q*.

Of course, in our models, every model stands for a kind of real networks, and every network can evolve to the last ideal state or balance state by the parameter modifications of *m*, *n*, *q*, *η*. Meanwhile, the sub-SF network *γ* ∈ (1, 2] and *γ* > 3 also contain a lot of very interesting conclusions and findings. In the future, we will continue to pay close attention to these special network states.

## Supporting Information

S1 File Program for data. The program data for Fig 2, which need to run by matlab.
(PDF)

## References

[1] Strogatz SH. Exploring complex network. Nature 2001 4(10):268–276. 10.1038/35065725 11258382

[2] Dorogovstsev SN, Mendes JFF. Evolution of networks. Adv. Phys. 2002(51):1079–1187. 10.1080/00018730110112519

[3] Costa L, Rodrigues FA, Travieso G. Characterization of complex networks: a survey of measurements. Adv. Phys. 2007(56):167–242. 10.1080/00018730601170527

[4] Luo C, Wang XY, Liu H. Controllability of time-delayed Boolean multiplex control networks under asynchronous stochastic update. Sci. Rep-UK 2014(4):7522 10.1038/srep07522 PMC4268650 25516009

[5] Boccaletti S, Latora V, Moreno Y, Chavez M, Hwang DU. Complex netwoks: structure and dynamics. Phys. Rep. 2006(424):175–308. 10.1016/j.physrep.2005.10.009

[6] Albert R, Barabási AL. Topology of evolving networks: local events and universality. Phys. Rev. Lett. 2000(85):5234–5237. 10.1103/PhysRevLett.85.5234 11102229

[7] Stefan B, Hans GS. Handbook of Graphs and Networks: From the Genome to the Internet. Wiley-VCH, Germany; 2003.

[8] Luo C, Wang XY, Liu H. Controllability of asynchronous Boolean multiplex control networks. Chaos 2014(24):033108 10.1063/1.4887278 25273188

[9] Erdös P, Rényi A. On the evolution of random graphs. Publ. Math Inst. Hungar. Acad. Sci. 1960(5):17–61.

[10] Watts DJ, Strogatz SH. Collective dynamics of small-world networks. Nature 1998(393):440–442. 10.1038/30918 9623998

[11] Barabási AL, Albert R. Emergence of scaling in random networks. Science 1999(286):509–512. 10.1126/science.286.5439.509 10521342

[12] May RM. Simple mathematical models with very complicated dynamics. Nature 1976(261):459–467. 10.1038/261459a0 934280

[13] Anishchenko A, Treves A. Autoassociative memory retrieval and spontaneous activity bumps in small-world networks of integrate-and-fire neurons. J. Physiol. Paris 2006(100)225–236. 10.1016/j.jphysparis.2007.01.004 17320359

[14] Amaral LAN, Scala A, Barthélémy M, Stanley HE. Classes of small-world networks. Proc. Natl. Acad. Sci. USA 2000(97):11149 10.1073/pnas.200327197 11005838 PMC17168

[15] Zhang LH, Li YJ, Wang M, Wang XJ, Xue SW. A novel deterministic hybrid complex network model created by inner-outer iteration. Nonlinear Dyn. 2012(69):1517–1523. 10.1007/s11071-012-0366-6

[16] Takemoto K, Akutsu T. Analysis of the effect of degree correlation on the size of minimum dominating sets in complex networks. PLoS ONE 2016(6):e0157868 10.1371/journal.pone.0157868 PMC4915616 27327273

[17] Fischer J, Kleidon A, Dittrich P. Thermodynamics of random reaction networks. PLoS ONE 2015(2): e0117312 10.1371/journal.pone.0117312 PMC4344194 25723751

[18] Liu Q, Fang JQ, Li Y. A unified dynamic scaling property for the unified hybrid network theory framework. Front. Phys. 2014(9):240–245. 10.1007/s11467-013-0389-6

[19] Cao H, Li Y. Unraveling chaotic attractors by complex networks and measurements of stock market complexity. Chaos 2014(24):013134 10.1063/1.4868258 24697396

[20] Niizato T, Gunji YP. Ongoing processes in a fitness network model under restricted resources. PLoS ONE 2015(5): e0127284 10.1371/journal.pone.0127284 PMC4436180 25985301

[21] Dorogovstsev SN, Mendes JFF. Evolution of networks with aging of sites. Phys. Rev. E 2000(62):1842–1845. 10.1103/PhysRevE.62.1842 11088645

[22] Bianconi G, Barabási AL. Competition and multiscaling in evolving networks. Europhys. Lett. 2001(54):436–442. 10.1209/epl/i2001-00260-6

[23] Papalexakis E, Hooi B, Pelechrinis K, Faloutsos C. Power-Hop: a pervasive observation for real complex networks. PLoS ONE 2016(3):e0151027 10.1371/journal.pone.0151027 PMC4790966 26974560

[24] Zhang LH, Chen J, Sun BL, Tang YY, Wang M, Li YJ, et al Nonlinear dynamic evolution and control in a new scale-free networks modeling. Nonlinear Dyn. 2014(76):1569–1578. 10.1007/s11071-013-1229-5

[25] Chen QH, Shi DH. The modeling of scale-free networks. Physica A 2004(335):240–248. 10.1016/j.physa.2003.12.014

