# Supplementary material for: Topology Universality and Dissimilarity in a Class of Scale-Free Networks
Source: PLoS One. 2016 Aug 25;11(8):e0161653. doi: 10.1371/journal.pone.0161653 (PMC4999222; doi:10.1371/journal.pone.0161653)
Supplement: S1 File — The program data for Fig 2, which need to run by matlab. (PDF) [file pone.0161653.s001.pdf]

```matlab
%The program data for Fig 2, which need to run by matlab
clear;
showplottool('plotbrowser');

subplot(1,2,1);%windows
%r=1.5
k=5:1:1000;
c=1000/1003*0.5*2^0.5;
z=(k-4).*sqrt(k-4);
y=c./z;
plot(k,y,'b-');
hold on;
%r=1.5
k=5:1:10;
a=0.5*2^0.5;
u=(k-4).*sqrt(k-4);
y=a./u;
plot(k,y,'g+');
k=8:5:20;
u=(k-4).*sqrt(k-4);
y=a./u;
plot(k,y,'g+');
k=15:10:100;
u=(k-4).*sqrt(k-4);
y=a./u;
plot(k,y,'g+');
k=70:50:300;
u=(k-4).*sqrt(k-4);
y=a./u;
plot(k,y,'g+');
k=220:100:1000;
u=(k-4).*sqrt(k-4);
y=a./u;
plot(k,y,'g+');
hold on;
%r=2
k=1:1:1000;
c=1000/1005*2;
z=(k-2).*(k-2);
y=c./z;
plot(k,y,'k-');
hold on;
%r=2
k=1:1:10;
a=2;
u=(k-2).*(k-2);
y=a./u;
plot(k,y,'r*');
k=8:5:20;
u=(k-2).*(k-2);
y=a./u;
plot(k,y,'r*');
k=15:10:100;
u=(k-2).*(k-2);
y=a./u;
plot(k,y,'r*');
k=70:50:300;
```

```
u=(k-2).*(k-2);
y=a./u;
plot(k,y,'r*');
k=220:100:1000;
u=(k-2).*(k-2);
y=a./u;
plot(k,y,'r*');
hold on;
%r=2.5
k=4:1:1000;
c=1000/1005*3/2;
z=(k-3).*(k-3).*sqrt(k-3);
y=c./z;
plot(k,y,'r-');
hold on;
%r=2.5
k=4:1:10;
a=1.5;
u=(k-3).*(k-3).*sqrt(k-3);
y=a./u;
plot(k,y,'ko');
k=8:5:20;
u=(k-3).*(k-3).*sqrt(k-3);
y=a./u;
plot(k,y,'ko');
k=15:10:100;
u=(k-3).*(k-3).*sqrt(k-3);
y=a./u;
plot(k,y,'ko');
k=70:50:300;
u=(k-3).*(k-3).*sqrt(k-3);
y=a./u;
plot(k,y,'ko');
k=220:100:1000;
u=(k-3).*(k-3).*sqrt(k-3);
y=a./u;
plot(k,y,'ko');
hold on;
%r=3
k=1:1:1000;
c=1000/1003*2*4;
z=k.*k.*k;
y=c./z;
plot(k,y,'g-');
hold on;
%r=3
k=1:1:10;
a=8;
u=k.*k.*k;
y=a./u;
plot(k,y,'md');
k=8:5:20;
u=k.*k.*k;
y=a./u;
plot(k,y,'md');
k=15:10:100;
u=k.*k.*k;
```

```
y=a./u;
plot(k,y,'md');
k=70:50:300;
u=k.*k.*k;
y=a./u;
plot(k,y,'md');
k=220:100:1000;
u=k.*k.*k;
y=a./u;
plot(k,y,'md');
hold on;
%r=3.5
k=6:1:1000;
c=1000/1010*2.5;
z=(k-5).*(k-5).*(k-5).*sqrt(k-5);
y=c./z;
plot(k,y,'m-');
hold on;
%r=3.5
k=6:1:10;
a=2.5;
u=(k-5).*(k-5).*(k-5).*sqrt(k-5);
y=a./u;
plot(k,y,'bv');
k=8:5:20;
u=(k-5).*(k-5).*(k-5).*sqrt(k-5);
y=a./u;
plot(k,y,'bv');
k=15:10:100;
u=(k-5).*(k-5).*(k-5).*sqrt(k-5);
y=a./u;
plot(k,y,'bv');
k=70:50:300;
u=(k-5).*(k-5).*(k-5).*sqrt(k-5);
y=a./u;
plot(k,y,'bv');
k=220:100:1000;
u=(k-5).*(k-5).*(k-5).*sqrt(k-5);
y=a./u;
plot(k,y,'bv');
hold on;

%legend('theoy','simulation',3);
text(500,0.1,'(a)');
%text(0.1,100,'- theory +* simulation');
xlabel('k');
ylabel('P(k)');
hold on;


subplot(1,2,2);
% ŋ =0.5
k=5:1:10;
A1=7/8*0.5;
X1=1/A1*(5+(-2)/A1)^1/A1;
Z1=X1./((k+(-2)/A1).^(1+1/A1));
```

```
plot(k,Z1,'r+');
hold on;
% ŋ =0.96
k=3:1:10;
A2=7/8*0.96;
X2=1/A2*(5+(-2)/A2)^1/A2;
Z2=X2./((k+(-2)/A2).^(1+1/A2));
plot(k,Z2,'k.');
hold on;
% ŋ =1.28
k=2:1:10;
A3=7/8*1.28;
X3=1/A3*(5+(-2)/A3)^1/A3;
Z3=X3./((k+(-2)/A3).^(1+1/A3));
plot(k,Z3,'b*');
hold on;
% ŋ =1.78
k=2:1:10;
A4=7/8*1.78;
X4=1/A4*(5+(-2)/A4)^1/A4;
Z4=X4./((k+(-2)/A4).^(1+1/A4));
plot(k,Z4,'m-');
hold on;

% ŋ =0.5
k=8:5:30;
Z1=X1./((k+(-2)/A1).^(1+1/A1));
plot(k,Z1,'r+');
k=25:10:99;
Z1=X1./((k+(-2)/A1).^(1+1/A1));
plot(k,Z1,'r+');
k=80:50:500;
Z1=X1./((k+(-2)/A1).^(1+1/A1));
plot(k,Z1,'r+');
k=420:100:1000;
Z1=X1./((k+(-2)/A1).^(1+1/A1));
plot(k,Z1,'r+');
% ŋ =0.96 ö
k=8:5:30;
Z2=X2./((k+(-2)/A2).^(1+1/A2));
plot(k,Z2,'k.');
k=25:10:99;
Z2=X2./((k+(-2)/A2).^(1+1/A2));
plot(k,Z2,'k.');
k=80:50:500;
Z2=X2./((k+(-2)/A2).^(1+1/A2));
plot(k,Z2,'k.');
k=420:100:1000;
Z2=X2./((k+(-2)/A2).^(1+1/A2));
plot(k,Z2,'k.');
% ŋ =1.28
k=8:5:30;
Z3=X3./((k+(-2)/A3).^(1+1/A3));
plot(k,Z3,'b*');
k=25:10:99;
Z3=X3./((k+(-2)/A3).^(1+1/A3));
plot(k,Z3,'b*');
```

```
k=80:50:500;
Z3=X3./((k+(-2)/A3).^(1+1/A3));
plot(k,Z3,'b*');
k=420:100:1000;
Z3=X3./((k+(-2)/A3).^(1+1/A3));
plot(k,Z3,'b*');
% ŋ =1.78
k=8:5:30;
Z4=X4./((k+(-2)/A4).^(1+1/A4));
plot(k,Z4,'m-');
k=25:10:99;
Z4=X4./((k+(-2)/A4).^(1+1/A4));
plot(k,Z4,'m-');
k=80:50:500;
Z4=X4./((k+(-2)/A4).^(1+1/A4));
plot(k,Z4,'m-');
k=420:100:1000;
Z4=X4./((k+(-2)/A4).^(1+1/A4));
plot(k,Z4,'m-');

legend('ŋ=0.5','ŋ=0.96','ŋ=1.28','ŋ=1.78',3);
text(500,0.1,'(b)');
xlabel('k');
ylabel('P(k)');
```
